# The P2Y_12_ Receptor Antagonist Ticagrelor Reduces Lysosomal pH and Autofluorescence in Retinal Pigmented Epithelial Cells From the ABCA4^-/-^ Mouse Model of Retinal Degeneration

**DOI:** 10.3389/fphar.2018.00242

**Published:** 2018-04-19

**Authors:** Wennan Lu, Néstor M. Gómez, Jason C. Lim, Sonia Guha, Ann O’Brien-Jenkins, Erin E. Coffey, Keith E. Campagno, Stuart A. McCaughey, Alan M. Laties, Leif G. Carlsson, Claire H. Mitchell

**Affiliations:** ^1^Department of Anatomy and Cell Biology, University of Pennsylvania, Philadelphia, PA, United States; ^2^Jules Stein Eye Institute, University of California, Los Angeles, Los Angeles, CA, United States; ^3^Department of Bioscience Heart Failure, Cardiovascular, Renal and Metabolic Diseases, IMED Biotech Unit, AstraZeneca, Gothenburg, Sweden; ^4^Department of Ophthalmology, University of Pennsylvania, Philadelphia, PA, United States; ^5^Department of Physiology, University of Pennsylvania, Philadelphia, PA, United States

**Keywords:** P2Y_12_ receptor, ticagrelor, age-related macular degeneration, lysosomal pH, retinal pigment epithelium, lysosomal storage diseases

## Abstract

The accumulation of partially degraded lipid waste in lysosomal-related organelles may contribute to pathology in many aging diseases. The presence of these lipofuscin granules is particularly evident in the autofluorescent lysosome-associated organelles of the retinal pigmented epithelial (RPE) cells, and may be related to early stages of age-related macular degeneration. While lysosomal enzymes degrade material optimally at acidic pH levels, lysosomal pH is elevated in RPE cells from the ABCA4^-/-^ mouse model of Stargardt’s disease, an early onset retinal degeneration. Lowering lysosomal pH through cAMP-dependent pathways decreases accumulation of autofluorescent material in RPE cells *in vitro*, but identification of an appropriate receptor is crucial for manipulating this pathway *in vivo*. As the P2Y_12_ receptor for ADP is coupled to the inhibitory G_i_ protein, we asked whether blocking the P2Y_12_ receptor with ticagrelor could restore lysosomal acidity and reduce autofluorescence in compromised RPE cells from ABCA4^-/-^ mice. Oral delivery of ticagrelor giving rise to clinically relevant exposure lowered lysosomal pH in these RPE cells. Ticagrelor also partially reduced autofluorescence in the RPE cells of ABCA4^-/-^ mice. *In vitro* studies in ARPE-19 cells using more specific antagonists AR-C69931 and AR-C66096 confirmed the importance of the P2Y_12_ receptor for lowering lysosomal pH and reducing autofluorescence. These observations identify P2Y_12_ receptor blockade as a potential target to lower lysosomal pH and clear lysosomal waste in RPE cells.

## Introduction

In some aging diseases, the accumulation of autofluorescent lipofuscin granules can signify an impaired clearance of waste material by lysosomes. Many degradative lysosomal enzymes are pH sensitive, with optimal activity in acidic environments. Lysosomal alkalinization has been detected in models of neural degenerative diseases of accumulation, such as early-onset Alzheimer’s disease (Lee et al., 2010, 2015; Coffey et al., 2014) and Stargardt’s retinal dystrophy (Liu et al., 2008).

Retinal pigmented epithelial (RPE) cells are particularly sensitive to perturbations in lysosomal enzyme activity, as they are responsible for phagocytosing the lipid-rich photoreceptor outer segment (POS) tips that are shed daily. The accumulation of autofluorescent lipofuscin waste may contribute to the early pathological changes leading age-related macular degeneration (AMD); dry geographic atrophy and wet neovascularization forms of AMD are now thought to stem from the intermediate AMD stage, where RPE cells are characterized by the accumulation of intracellular lipofuscin and extracellular drusen debris (Ferris et al., 2013). The accumulation of lipofuscin in RPE cells is associated with increases in the retinoid by-product *N*-retinylidene-*N*-retinylethanolamine (A2E), and A2E levels are increased in RPE cells from the ABCA4^-/-^ mouse model of recessive Stargardt’s retinopathy (Charbel Issa et al., 2013). Furthermore, A2E can lead to elevation of lysosomal pH, although the delay between drug application and alkalinization suggests an indirect pathway (Holz et al., 1999; Liu et al., 2008; Toops et al., 2015). This alkalinization may reduce lysosomal activity and contribute to a secondary accumulation of oxidized lipid waste.

Restoring an acidic environment to compromised lysosomes in RPE cells is predicted to enhance activity of pH-sensitive lysosomal enzymes and improve degradation, thus reducing the pathologies associated with accumulation of waste material (Guha et al., 2014a). While several pathways capable of acidifying compromised lysosomes and improving degradative function have been identified in RPE cells, manipulation of cytoplasmic cAMP was particularly effective (Liu et al., 2008). Drugs targeting receptors coupled to stimulatory G protein (G_s_) reduced lysosomal pH and enhanced the clearance of lysosomal waste and opsin turnover in RPE cells fed POSs (Liu et al., 2008; Guha et al., 2012). Importantly, this approach was also effective at lowering lysosomal pH when given to RPE cells isolated from ABCA4^-/-^ mice (Liu et al., 2012; Guha et al., 2014a). While these *in vitro* experiments provided proof of concept that drugs linked to cAMP could lower lysosomal pH and enhance lysosomal degradation, the *in vivo* translation of this approach required identification of the appropriate receptor target.

Several factors make the P2Y_12_ receptor antagonist ticagrelor (Brilinta) an attractive choice to target lysosomal accumulations in RPE cells. As the P2Y_12_ receptor for adenosine di-phosphate (ADP) is coupled to G_i_, antagonizing the P2Y_12_ raises cAMP (Cattaneo, 2015). Several P2Y_12_ receptor antagonists are widely used as antithrombotic agents and are approved for use in elderly patients (McFadyen et al., 2018). Ticagrelor is a reversible allosteric P2Y_12_ receptor antagonist that does not require hepatic activation, removing complications associated with genetic variants of the enzyme CYP2C19 common with other P2Y_12_ antagonists used clinically (Birkeland et al., 2010; Tantry et al., 2010). Ticagrelor is broadly utilized clinically to reduce the rate of thrombotic cardiovascular events in patients with acute coronary syndrome or a history of myocardial infarction (Storey et al., 2010; Bonaca et al., 2016). Finally, the P2Y_12_ receptor is expressed in cultured human ARPE-19 cells (Reigada et al., 2005). In this initial study, we examined whether ticagrelor lowers lysosomal pH and reduce lysosomal autofluorescence in RPE cells from the ABCA4^-/-^ mouse model of retinal degeneration.

## Materials and Methods

### Animal Care and Use

All procedures were approved by the University of Pennsylvania IACUC in compliance with the Public Health Service Policy on Humane Care and Use of Laboratory Animals. C57BL/6J and ABCA4^-/-^ mice were reared at 5–15 lux and sacrificed using CO_2_. C57BL/6J mice were obtained from Jackson Laboratories (Bar Harbor, ME, United States). ABCA4^-/-^ mice were obtained from Dr. Gabriel Travis of UCLA. All mice were negative for the RD8 mutation (Goméz et al., 2018). Mouse eyes were isolated and RPE cells processed as described previously (Liu et al., 2012).

### P2Y_12_ Receptor Pharmacological Agents

Ticagrelor was delivered in food or water at concentrations relevant to those used clinically in humans. The recommended maintenance dosage for ticagrelor (AZD6140) in humans of approximate mass of 90 kg is 180 mg per day; thus 2 mg/kg translated to 0.06 mg per diem for a 30 g mouse. Clinically dosed ticagrelor tablets (90 mg, Lot # YK0083 from the University of Pennsylvania pharmacy) were powdered and initially dissolved in water at 12 μg/ml to give 0.06 mg per diem, based on a mean water consumption of 5 ml per diem. (The concentration was adjusted to 10 μg/ml for later experiments to match the stated solubility more precisely, although precipitate was not detected in either concentration.) The solution was administered in tinted light-resistant bottles wrapped with black paper and refreshed every 1–2 days for 5–19 days. No clear correlation was found between exposure time or concentration, and lysosomal pH signal. Ticagrelor was delivered in food using a custom mouse diet containing 0.1% ticagrelor in Purina Lab Meal 5001 was made by MP Biomedicals (Lot #P9748, Santa Ana, CA, United States) from purified drug provided by AstraZeneca. Untreated food pellets or those containing 0.1% ticagrelor were added at 100–200 g every week and the remainder weighed to determine total food consumption. Ticagrelor has a pIC_50_ at the human P2Y_12_ receptor of 8.0 (Nylander and Schulz, 2016). The pIC_50_ of ticagrelor in an ADP-induced whole blood platelet aggregation assay in humans is 6.6, similar to that in mouse. Ticagrelor is reported to be quickly absorbed from the gut, reaching a peak concentration in 1.5 h, with blood plasma levels linearly dependent on the dose (Goel, 2013).

MeS-ADP; 2-(Methylthio)adenosine 5′-diphosphate (catalogue #1624, Tocris Bio-Techne Corporation, Minneapolis, MN, United States), which has a pIC_50_ of 8.2 and 7.9 at the P2Y_1_ and P2Y_12_ receptors, respectively (Jacobson et al., 2009), was supplied pre-dissolved in water at 10 mM and diluted. AR-C66931; *N*^6^-(2-methylthioethyl)-2-(3,3,3-trifluoropropylthio)-β,γ-dichloromethylene-ATP (a.k.a cangrelor, catalogue #5720 Tocris) with a pIC_50_ of 9.4 (Jacobson et al., 2009), was stored as a 10 mM stock solution in water. AR-C66096; 2-propylthio-betagamma-difluoromethylene ATP tetrasodium salt (catalogue #3321, Tocris) inhibits ADP-induced aggregation of washed human platelets pIC_50_ = 8.16 and was supplied pre-dissolved at a concentration of 10 mM.

### Measurement of Lysosomal pH From RPE Cells

Lysosomal pH was measured as described using the dye LysoSensor Yellow/Blue DND-160 (Liu et al., 2012). In brief, RPE cells from pairs of treated and untreated mice were isolated, loaded with LysoSensor Yellow/Blue 160 DSN, washed, and loaded into wells of a plate reader; the autofluorescence in these cells was previously found to be negligible in cells loaded with LysoSensor 160 DSN (Liu et al., 2008). The limited number of cells precluded calibration to absolute pH, so data were analyzed as the ratio of light excited at 340 vs. 380 nm, an index of lysosomal pH (Guha et al., 2014a). Lysosomal pH measurements were made with UV-Star 384-well plates (Grenier Bio One) to minimize the disruption of the signal at 340 nm. As these ratios can vary from experiment to experiment, values were normalized to enable results from multiple trials to be combined. Lysosomal pH was determined from cultured ARPE-19 cells as described (Guha et al., 2013).

### Polymerase Chain Reaction (PCR)

Total RNA was isolated from fresh mouse RPE/choroid cells using Trizol and the RNeasy mini kit (Qiagen, Inc.). RNA yield was determined by nanodrop 2000 spectrophotometer; 100 ng of total RNA was converted into cDNA using High Capacity RNA-to-cDNA kit (#4387406, Applied Biosystems). Primer pairs for mouse P2Y_12_: Forward: CATTGCTGTACACCGTCCTG; Reverse: AACTTGGCACACCAAGGTTC; 212-bp product. PCR was performed with 2 μl first-strand DNA synthesis product, 50 mM MgCl_2_, and10 μM of each primer with the 0.5 μl first recombinant DNA polymerase (Platinum^®^ *Taq* DNA Polymerase; Applied Biosystems) at 95°C for 10 min, followed by 35 cycles at 95°C for 30 s, 60°C for 45 s, and 72°C for 1 min, with a final extension step at 72°C for 10 min. First-strand DNA synthesis was omitted from the negative control. Quantitative PCR (qPCR) was performed on isolated RPE/choroid from 16 month old C57BL6J or ABCA4^-/-^ mice. Total RNA (100 ng) was reverse transcribed and qPCR was performed using SYBR Green and the 7300 RealTimePCR system (Applied Biosystems, Corp.) as described (Lu et al., 2017) using the following primers: A1AR- F: ATCCCTCTCCGGTACAAGACAGT, R: ACTCAGGTTGTTCCAGCCAAAC (Streitova et al., 2010); A3AR- F: ACTTCTATGCCTGCCTTTTCATGT, R: AACCGTTCTATATCTGACTGTCAGCTT (Streitova et al., 2010); CFH- F:ACCACATGTGCCAAATGCTA; R:TGTTGAGTCTCGGCACTTTG (Radu et al., 2014); ENT1- F:CTTGGGATTCAGGGTCAGAA, R: ATCAGGTCACACGACACCAA (Eckle et al., 2013); P2Y12- F: CATTGCTGTACACCGTCCTG, R: AACTTGGCACACCAAGGTTC (Veitinger et al., 2011). Data were analyzed using the delta-delta CT approach, with results expressed as fold change in gene expression.

### Microscopy and Immunocytochemistry

Freshly isolated RPE cells from C57Bl6J mice were cultured for 1 day and fixed in 4% paraformaldehyde (PFA), rinsed with Duebcco’s phosphate buffered saline (DPBS), permeabilized at room temperature in 0.1% Triton X-100 for 10 min, then blocked with 10% goat serum in SuperBlock blocking buffer (#37515, Thermo Fisher Scientific) for 60 min. Anti- mouse P2Y_12_ antibody (1:50 dilution, #AS-55043A, AnaSpec, Inc., Fremont, CA, United States) was added overnight at 4°C, followed by incubation in donkey anti-rabbit Alexa-Fluor 568 (1:500 dilution; Invitrogen, Carlsbad, CA, United States). Slides were mounted in Slow Fade Gold Antifade Mountant (Thermo Fisher Scientific) and imaged using a Nikon Eclipse 600 microscope (Nikon USA, Melville, NY, United States). To quantify the autofluorescence found in RPE whole mounts from ABCA4^-/-^ mice, images were obtained at 488 nm ex/>540 nm em from 16 regions using a Nikon Eclipse 600 microscope and autofluorescence was measured using the Nikon Elements software. Spectral analysis of RPE cells in ABCA4^-/-^ mice was performed on 7 μm thick sections from the central 1/3^rd^ of the retina following standard fixation (Albalawi et al., 2017) using the Nikon A1R Laser Scanning Confocal Microscope and Nikon NIS-Elements software package at the University of Pennsylvania Live Cell Imaging Core. The endogenous autofluorescent spectral profile of ABCA4^-/-^ mouse RPE was collected after excitation by 406, 488, 561, and 639 lasers. The RPE layer was visualized with a 60X objective and emission was determined in 2.5 nm wide bins throughout the spectrum, grouped into 32 bins per sweep. Confocal scan settings were maintained from sample to sample, and pairs of treated and untreated sections were processed in parallel when possible to minimize day-to-day variations. ROI’s were drawn along the RPE layer and corresponding emission levels were exported for analysis.

### ARPE-19 Cell Culture

ARPE-19 cells (American Tissue Type Collection, Manassas, VA, United States) were grown as described previously (Reigada et al., 2005). In brief, cells were grown in 1:1 mixture of Dulbecco’s modified Eagle’s medium (DMEM) and Ham’s F12 medium with 3 mM L-glutamine, 100 g/ml streptomycin and 10% FBS (all Thermo Fisher Scientific, Inc., Waltham, MA, United States). Cells were incubated at 37°C in 5% CO_2_ and subcultured weekly with 0.05% trypsin and 0.02% ethylenediaminetetraacetic acid (EDTA).

### *In Vitro* Autofluorescence Model

ARPE-19 cells were grown to confluence in 6-well plates, then treated with the pulse chase chloroquine/POS protocol as described (Guha et al., 2012). Cells were incubated with 2 ml POS for 2 h; POS were isolated as previously described (Liu et al., 2012). Cells were washed thoroughly with medium to remove non-internalized POS followed by a 2 h chase in DMEM/F12. Subsequently, the medium was removed and the cells incubated for 20 h with one of the following solutions: DMEM/F12, 10 μM CHQ, CHQ + 10 μM AR-C66096. This protocol was repeated daily. After 6 days the cells were repeatedly washed, detached with trypsin, and analyzed on a flow cytometer (FACS Calibur; BD Biosciences, Heidelberg, Germany) at the Penn PDM Flow Cytometry Facility. The FITC channel (excitation laser wavelength, 488 nm; detection filter wavelength, 530 nm) was used with a gate set to exclude cell debris and cell clusters.

### Magic Red Staining

Magic Red powder was reconstituted as per the manufacturer’s instructions (Bio-Rad, Inc., Hercules, CA, United States). ARPE-19 cells grown on coverslips were exposed to 10 μM tamoxifen or control solution. Magic Red was diluted into PBS 1:26 and applied to the cells for 30 min, followed by a 5 min incubation of 50 nM LysoTracker Green. Magic Red was visualized at 540 nm ex and LysoTracker Green at 488 nm using the Nikon Eclipse 600, as above.

### Data Analysis

All data are given as mean ± standard error of the mean. Analysis was performed using SigmaStat (Systat Software, Inc., San Jose, CA, United States) and/or GraphPad Software, Inc. (La Jolla, CA, United States). Differences between treatments were analyzed using a one-way analysis of variance (ANOVA) with indicated *post hoc* tests as appropriate, or a Student’s *t*-test using unpaired or paired configuration where appropriate.

## Results

### Systemic Delivery of P2Y_12_ Antagonist Ticagrelor Lowers Lysosomal pH in RPE Cells From ABCA4^-/-^ Mice

Initial experiments to determine whether ticagrelor decreased lysosomal pH in RPE cells from ABCA4^-/-^ mice were carried out by adding ticagrelor to the drinking water. Mice were given free access to drinking water only or water containing 10–12 μg/ml ticagrelor for 4–19 days. Fresh solution was provided every 1–2 days. Measurements of remaining water suggested ticagrelor did not alter consumption. Age- and gender-matched ABCA4^-/-^ mice used for this experiment examined daily appeared healthy and exhibited normal behavior with no increased bleeding events noted. Previous work suggest lysosomal pH must be measured from freshly isolated RPE cells from ABCA4^-/-^ mice, as the effect of lipofuscin on the lysosomal pH is altered by each cell division (Guha et al., 2014a). As such, lysosomal pH levels could only be determined accurately from only pair of one untreated and one treated mouse per day.

**FIGURE 1:**
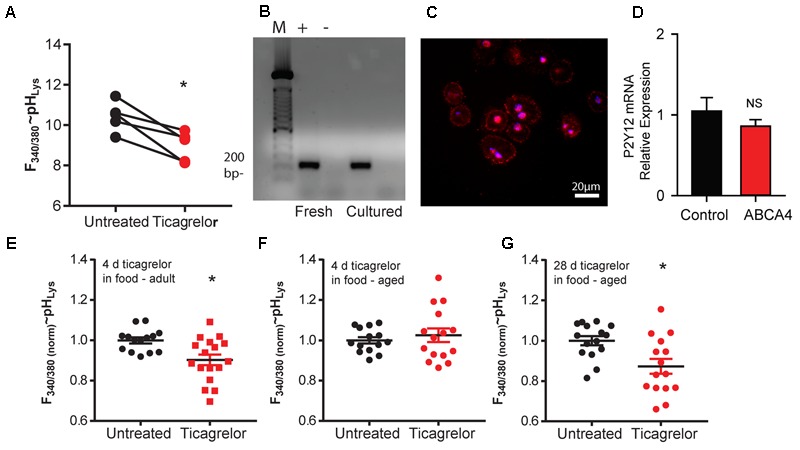
Ticagrelor lowers lysosomal pH in ABCA4^-/-^ mice. **(A)** Decline in the ratio of LysoSensor YellowBlue DNS 160 fluorescence excited at 340 vs. 380 nm in freshly isolated RPE cells from 20-month old untreated ABCA4^-/-^ mice (black circles) and those treated with 10–12 μg/ml ticagrelor in water (red squares). As mice were processed in pairs in individual days, lines connect mean measurements from treated and untreated pairs processed in parallel. ^∗^*p* = 0.029, *n* = 5, each circle represents the mean of 5–6 wells. **(B)** RT-PCR (+) shows P2Y_12_ receptor expression in RPE cells freshly isolated from mouse eyes (Fresh) or after 3 days in cell culture (Cultured). Bands are at the expected size of 212 bp. No product was detected when reverse transcriptase was omitted from the reaction (–). M-markers at 100 bp (see Supplementary Figure 3 for full gel). **(C)** Immunohistochemistry indicating expression of P2Y_12_ near the cell membrane of freshly isolated RPE cells from 9-month-old C57BL/6J mice. **(D)** Results from quantitative PCR assessment indicating there is no significant (NS) difference between expression of P2Y_12_ receptor mRNA from RPE/choroid of C57BL/6J (*n* = 4) and ABCA4^-/-^ mice (*n* = 3). **(E)** Ticagrelor added to a custom diet (0.1%) for 4 days was sufficient to lower lysosomal pH in RPE cells from ABCA4^-/-^ mice 7–8 months old (adult) (^∗^*p* = 0.0054, *n* = 14–17 measurements from three mice each condition). **(F)** Exposure to ticagrelor in food for 4 days did not reduce the mean signal in RPE cells from ABCA4^-/-^ mice aged 18–24 months (Old) (*p* = 0.514, *n* = 14–15 measures from three mice each condition). **(G)** Extending ticagrelor treatment to 28 days was sufficient to reduce lysosomal pH in the older mice (^∗^*p* = 0.0066, *n* = 15 measures from three mice each condition). Throughout, data are expressed as scatter plot with mean ± SEM of the ratio of light excited at 340/380 nm; values were normalized to the mean control for each day to account for differences in LysoSensor dye loading.

Ticagrelor acidified the lysosomes of RPE cells from ABCA4^-/-^ mice. The lysosomal pH signal was reduced in RPE cells from treated mice as compared to untreated mice in all pairs examined, leading to a significant decline when averaged together (**Figure 1A**). The presence of the P2Y_12_ receptor in mouse RPE cells was confirmed at the mRNA level (**Figure 1B**) and using immunocytochemistry; expression was concentrated near the cell membrane consistent with a membrane-associated receptor (**Figure 1C**). Quantitative PCR indicated no difference in the expression of P2Y_12_ mRNA in RPE cells from C57BL/6J and ABCA4^-/-^ mice (**Figure 1D**).

In a separate study, ticagrelor was added to the mouse chow to confirm the ability of oral delivery to lower lysosomal pH in the RPE cells of ABCA4^-/-^ mice. Four days of exposure lowered the lysosomal pH in 7–8 month-old adult ABCA4^-/-^ mice, as compared to untreated mice (**Figure 1E**). Four days of treatment did not alter the lysosomal pH of RPE cells from 18 to 24 month-old mice compared to untreated controls (**Figure 1F**), but extending treatment to 28 days significantly reduced lysosomal pH in these older mice (**Figure 1G**). Plasma levels of ticagrelor in the mice treated with ticagrelor in food were 0.89 ± 0.07 μM (*n* = 13) and were undetectable in untreated mice.

### P2Y_12_ Receptor Regulates Lysosomal pH *in Vitro*

**FIGURE 2:**
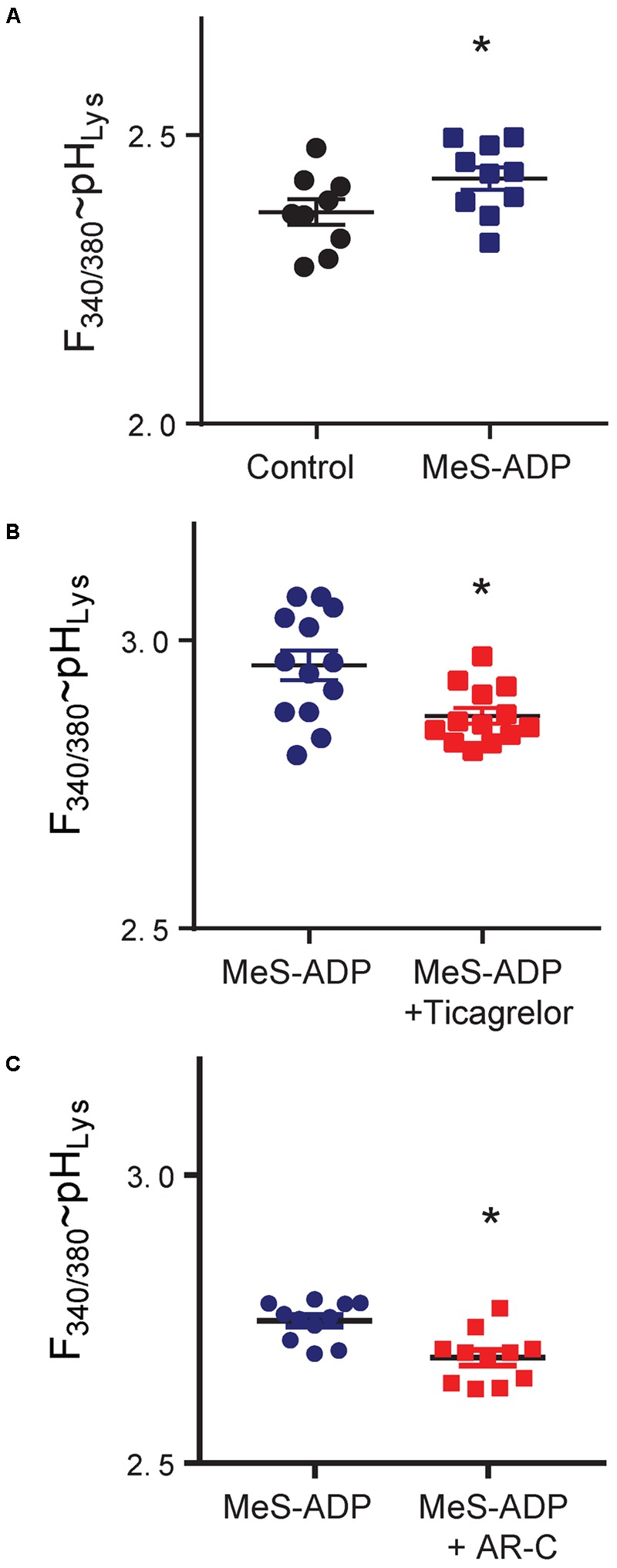
Manipulation of the P2Y_12_ receptor alters lysosomal pH in ARPE-19 cells. **(A)** The lysosomal pH of ARPE-19 cells was elevated by P2Y_12_ receptor agonist methylthio ADP (MeS-ADP, 10 μM). ^∗^*p* = 0.002, *n* = 9–10. **(B)** Ticagrelor (20 μM) lowered the lysosomal pH in cells treated with MeS-ADP (^∗^*p* = 0.006, *n* = 13). **(C)** The P2Y_12_ receptor antagonist AR-C66931 (ARC, 10 μM) also reduced lysosomal pH in ARPE-19 cells treated with MeS-ADP (^∗^*p* = 0.033, *n* = 10). Data are expressed as the ratio of fluorescence excited at 340 vs. 380 nm in cells loaded with LysoSensor Yellow/Blue DNS 160; this is indicative of lysosomal pH, although absolute ratios varied with preparation; n represents the number of wells in a single trial with figures representative of 2–4 trials.

The ability of ticagrelor to lower lysosomal pH was tested directly *in vitro* in the ARPE-19 cultured human cell line. The P2Y_12_ agonist Mes-ADP raised lysosomal pH in these cells (**Figure 2A**). Addition of ticagrelor (**Figure 2B**) or P2Y_12_ receptor antagonist AR-C66931 (**Figure 2C**) to the ARPE-19 cells reduced the lysosomal pH relative to MeS-ADP alone.

### P2Y_12_ Receptor Antagonists Reduce Autofluorescence

**FIGURE 3:**
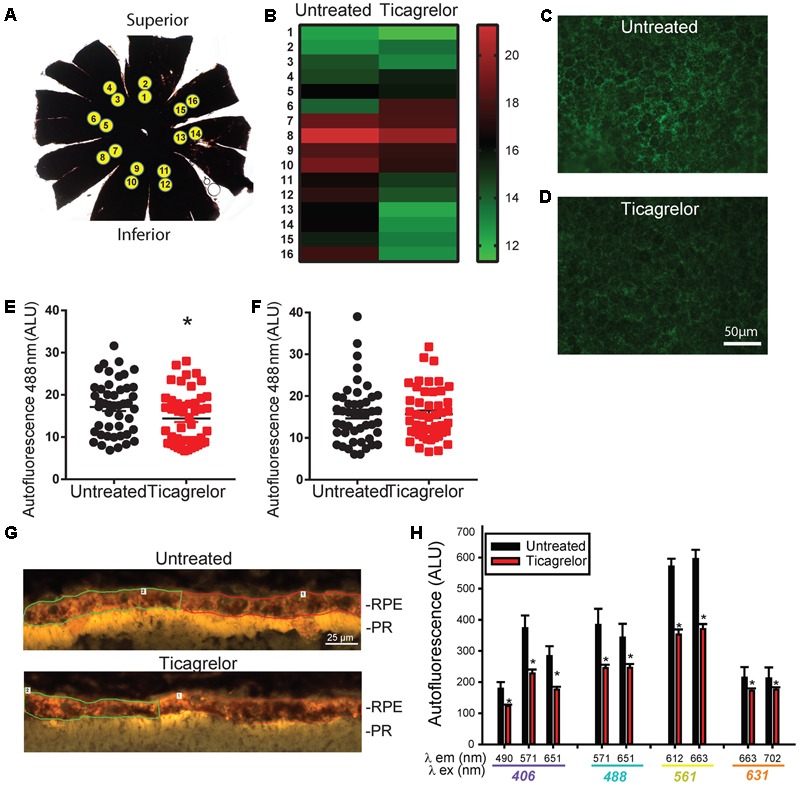
Ticagrelor reduces autofluorescence in RPE cells from ABCA4^-/-^ mice. **(A)** A RPE whole mount preparation indicating the location of images obtained for autofluorescence quantification. **(B)** Heat map illustrating variation in intensity of autofluorescence (488 nm ex/>525 nm em) in regions corresponding to those in “A.” Each band is the mean of six untreated mice or mice treated with 0.1% ticagrelor in food for 28 days. Scale at right shows relative intensity. Images of autofluorescence excited at 488 nm in inferior regions of RPE wholemounts from untreated **(C)** and treated **(D)** ABCA4^-/-^ mice. **(E)** Autofluorescence intensity from regions 9–16 of six mice in each condition (*p* = 0.039, *n* = 48 measurements; eight from each mouse). **(F)** Autofluorescence intensity from regions 1–8 of six mice in each condition, *n* = 48). Mice were 257–333 days old after ticagrelor treatment. **(G)** Images from sections of the outer retina showing the RPE and photoreceptor outer segments (POSs) in untreated 20 month old ABCA4^-/-^ mice at 406 nm ex/>409 nm em. The demarcation of two regions of interest (ROI) in the RPE layer are shown. Analogous image for 20 month old ABCA4^-/-^ mouse treated with 10 μg/ml ticagrelor in drinking water for 4 days. **(H)** Mean autofluorescence output for key excitation/emission pairs wavelengths (λ, as indicated; ^∗^*p* < 0.05, *n* = 16 untreated, 10 treated sections from 4 to 5 mice respectively).

Lowering lysosomal pH increased clearance of autofluorescent material in prior *in vitro* work (Liu et al., 2008, 2012; Baltazar et al., 2012; Guha et al., 2012). To determine whether ticagrelor could decrease autofluorescence *in vivo*, levels were determined from 16 regions of the RPE whole mount from untreated mice and those receiving ticagrelor in food (**Figure 3A**). There was considerable variation in the autofluorescence values in untreated mice. However, comparing the pattern of autofluorescence across all regions suggested there was a greater difference in autofluorescent levels in samples from the inferior/nasal region (**Figure 3B**). Representative images show the autofluorescence in untreated (**Figure 3C**) and treated mice (**Figure 3D**). Quantification indicated that the autofluorescence from RPE cells in the interior/nasal regions was reduced (**Figure 3E**) in mice treated with ticagrelor. However, there was no difference in mean levels in the superior/temporal regions (**Figure 3F**).

To further characterize the effect of ticagrelor, autofluorescence in RPE cells was analyzed in retinal sections from untreated ABCA4^-/-^ mice and mice treated with ticagrelor. Increased autofluorescence in the RPE cells was detected in many sections from the untreated mice, as compared to those treated with ticagrelor (**Figure 3G**). While variation in autofluorescence levels across regions and mice was considerable, quantification of the autofluorescent output for a broad range of excitation/emission pairs showed a reduction, with emission at 571, 612, and 663 nm particularly effected (**Figure 3H**).

### P2Y_12_ Receptor Antagonism Reduces Autofluorescence *in Vitro*

**FIGURE 4:**
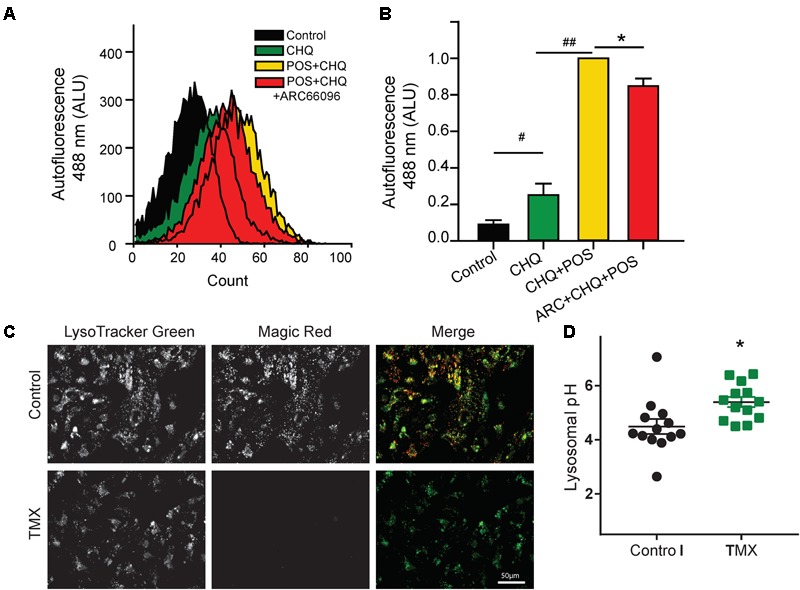
Blocking P2Y_12_ reduces RPE autofluorescence *in vitro.* **(A)** Treatment of ARPE-19 cells with POSs and 10 μM chloroquine (CHQ) using the pulse chase method led to a substantial rise in autofluorescence at 488 nm ex/>525 nm em. FACS analysis demonstrates the increased autofluorescence with the CHQ/POS treatment for >6 days. Treatment of cells with 10 μM P2Y_12_ receptor antagonist AR-C66096 shifted the autofluorescence curve to the left. **(B)** Quantification of the mean autofluorescence from five independent FACS trials showing a significant reduction in POS/CHQ autofluorescence excited at 488 nm in cells treated with 10 μM AR-C66096 (#*p* = 0.02, ##*p* < 0.001, ^∗^*p* = 0.014, *n* = 5). **(C)** ARPE-19 cells displaying overlap of Magic Red and LysoTracker Green, suggesting cathepsin B activity in lysosomes under control conditions (top). Magic Red staining was eliminated by elevation of lysosomal pH with 10 μM tamoxifen (bottom). **(D)** Tamoxifen (10 μM) raises lysosomal pH in ARPE-19 cells (^∗^*p* = 0.012, *n* = 13).

The effect of P2Y_12_ antagonism on autofluorescence was determined *in vitro* using FACS analysis following application of the pulse chase protocol for loading ARPE-19 cells with POSs and alkalinizing lysosomes with chloroquine (see section “Materials and Methods”). Cellular autofluorescence was increased by chloroquine alone, but the addition of POSs increased this autofluorescence substantially (**Figure 4A**). The P2Y_12_ receptor antagonist AR-C66096 reduced the autofluorescence produced by POSs in cells treated with chloroquine (**Figure 4B**). This suggests that blocking the P2Y_12_ receptor reduces lipofuscin accumulation in RPE cells *in vitro*, as it does *in vivo*.

To strengthen the link between lysosomal pH and degradative enzyme activity, ARPE-19 cells were stained with Magic Red to determine cathepsin B activity. The Magic Red substrate releases fluorescent cresyl violet in organelles containing cathepsin B that is catalytically active (Creasy et al., 2007). Magic Red staining was substantial under control conditions; staining colocalized LysoTracker Green, consistent with the lysosomal localization of cathepsin B activity (**Figure 4C**). To determine whether rapid changes in lysosomal pH alter the activity of cathepsin B, cells were treated with tamoxifen, which raises lysosomal pH more rapidly and reproducibly than chloroquine in these cells (**Figure 4D**, Liu et al., 2008). Magic Red staining was absent in cells treated with tamoxifen, consistent with the pH dependence of degradative enzymes in ARPE-19 cells.

### Ticagrelor and Gene Expression

The effect of ticagrelor on expression of several key genes in RPE cells was examined. Relative gene expression analysis using quantitative PCR showed a significant downregulation in the expression of the equilibrative nucleoside transporter 1 (ENT1), and complement factor H (CFH) in the RPE of mice treated with 0.1% ticagrelor in food for 14 days. There was no change in expression of mRNA for the P2Y_12_ receptor, the A_1_ adenosine receptor or the A_3_ adenosine receptor (Supplementary Figure 1).

## Discussion

We found that systemic delivery of ticagrelor lowered lysosomal pH in RPE cells of ABCA4^-/-^ mice. Lysosomal pH was reduced both by ticagrelor added to the drinking water, and by addition of ticagrelor to the mouse chow. Results from ticagrelor in food suggest older mice require longer treatment for lysosomal acidification. Ticagrelor, as well as the P2Y_12_ antagonist AR-C66931, lowered lysosomal pH in cultured human ARPE-19 cells, showing receptor block had a direct effect on lysosomal pH in RPE cells. Ticagrelor delivered orally also reduced autofluorescence in the inferior/nasal regions of these RPE cells, while block of the P2Y_12_ receptor reduced autofluorescence *in vitro* in ARPE-19 cells fed POSs. This provides the first evidence that systemic delivery of a P2Y_12_ antagonist improves lysosomal dysregulation in RPE cells.

### Contribution of the P2Y12 Receptor

Several observations implicate block of the P2Y_12_ receptor in the lysosomal acidification by ticagrelor. Previous studies indicated that elevation of cAMP, either directly or through drugs known to target the G_s_ protein that stimulates adenylate cyclase, lowered lysosomal pH in RPE cells from ABCA4^-/-^ mice when applied *ex vivo*, and in compromised ARPE-19 cells (Liu et al., 2008, 2012; Guha et al., 2012, 2014b). This lysosomal acidification was blocked by a protein kinase inhibitor (PKI), implicating protein kinase A in the acidification. As the P2Y_12_ receptor is coupled to the inhibitory G_i_ protein, P2Y_12_ receptor antagonists will produce similar effects (Supplementary Figure 2). Although ticagrelor can also inhibit the equilibrative nucleoside transporter 1 to raise extracellular adenosine (Nylander et al., 2013; Armstrong et al., 2014; Aungraheeta et al., 2016), it is unlikely adenosine contributes much to the response in this study as plasma levels of ticagrelor in our treated mice were 0.87 ± 0.07 μM, while plasma levels of adenosine reached half -maximal concentrations with ∼30 μM ticagrelor (Nylander et al., 2013). In addition, AR-C66931 does not act on ENT1 (Armstrong et al., 2014), and thus the ability of AR-C66931 to acidify lysosomes *in vitro* indicates P2Y_12_ antagonism is sufficient to lower lysosomal pH in these cells. Together, this implicates the P2Y_12_ receptor in mediating the effects of ticagrelor observed in this study.

### Mechanisms for Reduced Autofluorescence

Ticagrelor may reduce autofluorescence from RPE cells through multiple pathways. For example, the lysosomal pH in RPE cells from ABCA4^-/-^ mice is alkalinized above age-matched controls (Liu et al., 2008) and lowering lysosomal pH with ticagrelor would enhance the activity of pH sensitive degradative lysosomal enzymes. Data above using Magic Red indicate cathepsin B activity in RPE cells is decreased by moderate elevations in lysosomal pH; this parallels the rise in cathepsin D activity following lysosomal acidification in RPE cells found previously (Guha et al., 2012). Acidifying lysosomal pH in RPE cells also enhanced the turnover of opsin derived from phagocytosed POSs, suggesting pH manipulation can enhance turnover of phagocytosed waste (Baltazar et al., 2012).

In addition to modulating the activity of lysosomal enzymes, decreased autofluorescence in RPE cells may reflect an enhanced exocytosis of waste material. The *bis*-retinoid A2E is present in high levels in the RPE cells of ABCA4^-/-^ mice (Mata et al., 2001). Although the endogenous breakdown of A2E is difficult (Wu et al., 2011), A2E shows a peak autofluorescence emission at 570 nm when excited at 380 nm (Sparrow et al., 1999), and the decrease in this emission in mice receiving ticagrelor suggests the A2E may be exocytosed. The TRPML1 channel contributes to the exocytosis of lysosomal waste; TRPML1 activity is pH dependent (Samie et al., 2013; Li et al., 2017), and recent studies suggest the TRPML1 channel is particularly active in RPE cells (Goméz et al., 2018). It is possible that enhanced exocytosis may contribute to the reduced autofluorescence in ABCA4^-/-^ mice receiving ticagrelor, although further experiments are needed to confirm this.

### Remaining Issues

Several additional issues remain unresolved in this study. For example, it is not clear why elderly ABCA4^-/-^ mice required an extended treatment with ticagrelor before lysosomal pH was reduced, while there was no detectable trend between treatment length and the lysosomal acidification in mice receiving ticagrelor in their drinking water, especially as plasma concentrations of ticagrelor did not increase with prolonged dosage. Furthermore, it is unclear why the decline in RPE autofluorescence was limited to the inferior/nasal regions of the whole mounts. Regional differences in photoreceptor death occur with light damage models, with degree of light and differential levels of rhodopsin implicated (Organisciak and Vaughan, 2010); both of these parameters could influence the clearance of autofluorescence by ticagrelor. The mechanisms by which ticagrelor can decrease expression of CFH and ENT1 are also unclear; neither gene is linked to the CLEAR network directly regulated by lysosomal activity suggesting a more indirect connection (Palmieri et al., 2011). As discussed above, the relative effects of ticagrelor on lysosomal degradation versus exocytosis of autofluorescent waste are also relevant. Future work is needed to address these issues.

### Relevance to Human Doses

Given that ticagrelor is used in mainly elderly patients, the comparison of doses used here in mice with human dosage is relevant. Plasma levels in mice receiving 0.1% ticagrelor in food were 0.89 μM; this corresponds to 465 ng/mL and is close to levels in mice reported recently on 0.1% ticagrelor (Preusch et al., 2016). In humans receiving the standard dose of 180 mg/day, plasma concentrations of ticagrelor ranged from a maximum of 770 ng/ml 2 h after dosing to a minimum of 227 ng/mL (Storey et al., 2007, 2016). This suggests the levels of ticagrelor that lower lysosomal pH and decrease autofluorescence in mice are within the range found in patients. Whether treatments with ticagrelor can protect vision in ABCA4^-/-^ mice is currently being evaluated.

Portions of this work have previously been presented in abstract form (Lu et al., 2017).

## Ethics Statement

Ethics Approval and Consent to Participate: All experimental approaches on mice were approved by the Animal Care and Use Committee of the University of Pennsylvania protocol #804588.

## Availability of Data and Materials

All readily reproducible materials described in the manuscript, including new software, databases and all relevant raw data will be freely available to any scientist wishing to use them.

## Author Contributions

WL, NG, JL, SG, EC, AO, and KC performed the experiments. SM, LC, AL, and CM wrote the manuscript. CM and AL conceived of the idea.

## Conflict of Interest Statement

AstraZeneca partially supported this work through a granting mechanism. LC is employed by AstraZeneca, CM and AL are associated with intellectual property related to this topic. The other authors declare that the research was conducted in the absence of any commercial or financial relationships that could be construed as a potential conflict of interest.
