# Supplementary figures and images for: The P2Y12 Receptor Antagonist Ticagrelor Reduces Lysosomal pH and Autofluorescence in Retinal Pigmented Epithelial Cells From the ABCA4-/- Mouse Model of Retinal Degeneration
Source: Front Pharmacol. 2018 Apr 19;9:242. doi: 10.3389/fphar.2018.00242 (PMC5917064; doi:10.3389/fphar.2018.00242)

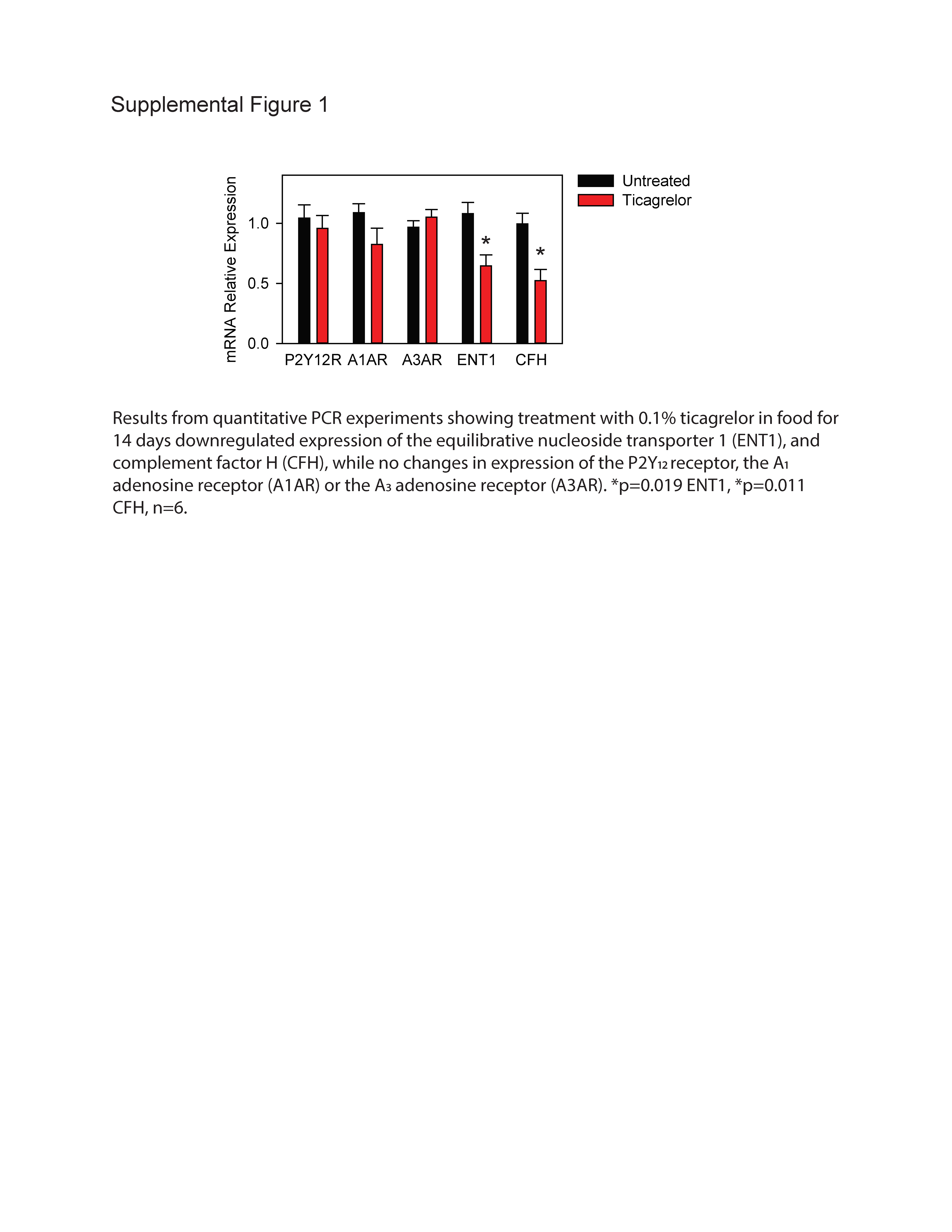

Supplement: Supplementary file 1 [file Image_1.TIF]

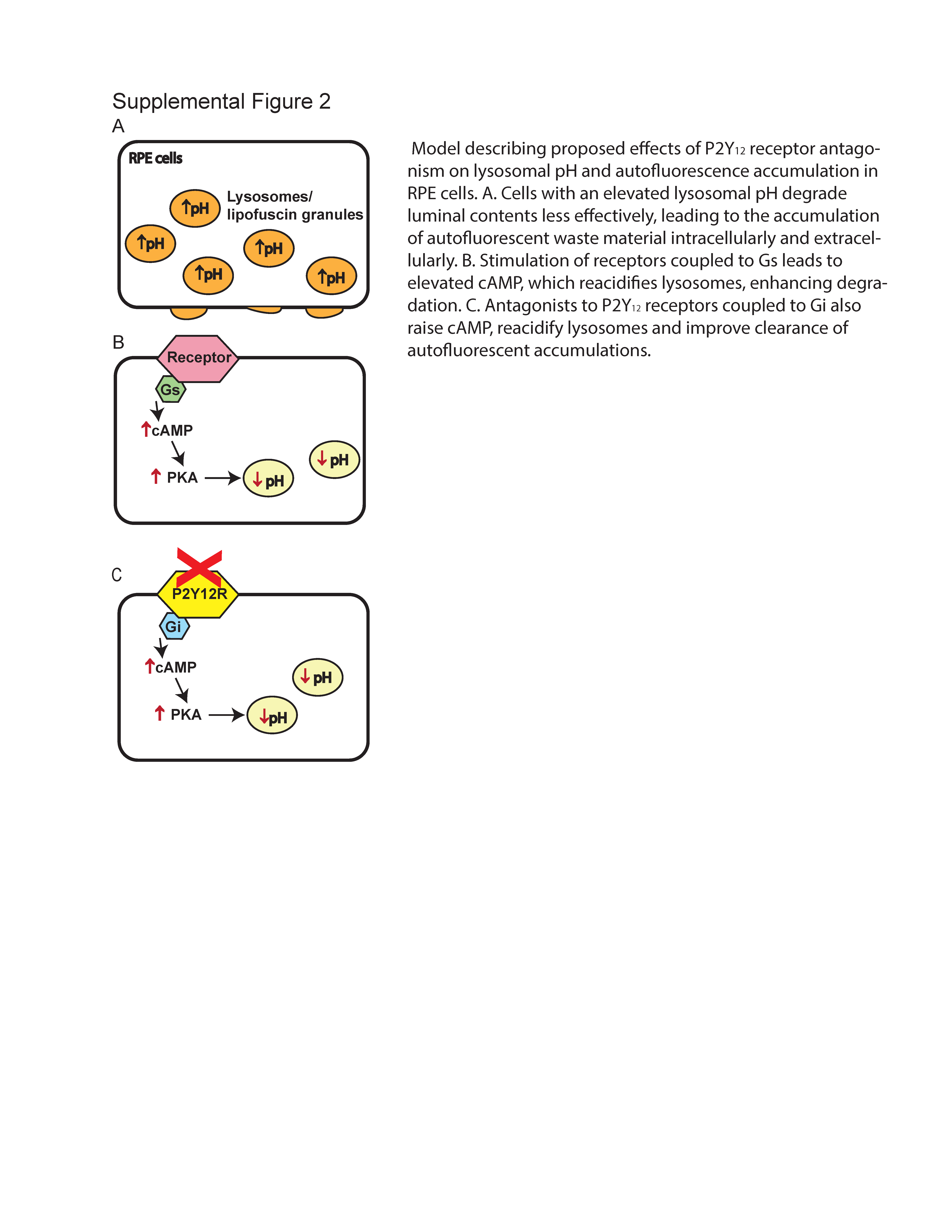

Supplement: Supplementary file 2 [file Image_2.TIFF]

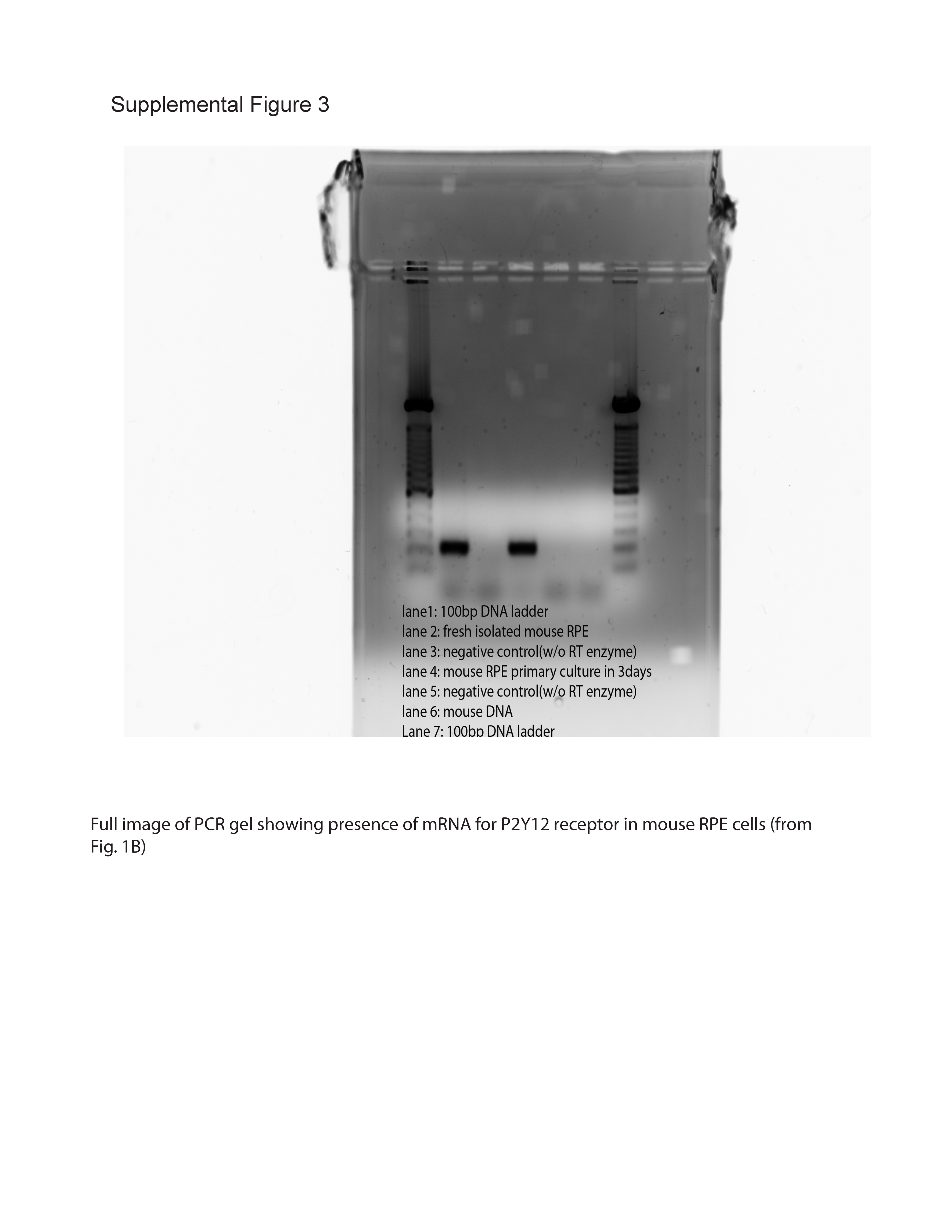

Supplement: Supplementary file 3 [file Image_3.TIF]
